# Response to “An exceptionally preserved 110 million years old praying mantis provides new insights into the predatory behaviour of early mantodeans”

**DOI:** 10.7717/peerj.4046

**Published:** 2017-11-16

**Authors:** Sydney K. Brannoch, Gavin J. Svenson

**Affiliations:** 1 Department of Invertebrate Zoology, Cleveland Museum of Natural History, Cleveland, OH, United States of America; 2 Department of Biology, Case Western Reserve University, Cleveland, OH, United States of America

**Keywords:** Mantodea, Praying mantis, Raptorial appendage, Cursorial appendages, Predatory behavior, Fossil, *Santanmantis axelrodi*, Rebuttal, Behavior

## Abstract

Hörnig, Haug & Haug (2017) published a description of a new specimen of *Santanmantis axelrodi* MB.I.2068, an extinct species of praying mantis from the Crato Formation of Brazil. According to Hörnig, Haug & Haug (2017), the discovery of this new specimen brought with it implications for praying mantis character evolution and predatory behavior; it is with these lines of reasoning that we find fault. More specifically, we point to four flawed assumptions in their study that led to their unsubstantiated conclusion that *S. axelrodi* employed their mesothoracic legs in prey capture.

## Introduction

Hörnig, Haug & Haug (2017) describe an incomplete fossil specimen of *Santanmantis axelrodi* Grimaldi, 2003 (MB.I.2068) from the Crato Formation in Brazil. *Santanmantis axelrodi* is regarded as an early (Hörnig, Haug & Haug, 2013; Hörnig, Haug & Haug, 2017) or primitive (Grimaldi, 2003; Lee, 2014) species of praying mantis (Insecta, Mantodea) armed with spine-laden raptorial forelegs. Raptorial forelegs with forefemoral brushes are considered to be autapomorphic for Mantodea (Klass & Ehrmann, 2003; Wieland, 2013), and in extant species consist of a foretibiae that can close against the forefemora to ensnare prey (Wieland, 2013). Due to the relatively more complete preservation of the dextral mesothoracic femur and tibia of this *S. axelrodi* specimen compared to others, Hörnig, Haug & Haug (2017) were able to describe the morphology, specifically the spination, of the mesothoracic leg in greater detail and conclude that the spination resembles that of the foreleg spination in “rigidity, shape, length, orientation and pointedness.” According to Hörnig, Haug & Haug (2017), the discovery of this new fossil specimen with a more completely preserved mesothoracic appendage brought with it implications for mantodean character evolution and predatory behavior. It is with these lines of reasoning that we find fault. More specifically, we point to four assumptions in their study that led to their unsubstantiated conclusion that *S. axelrodi* employed their mesothoracic legs in prey capture. These include: (1) the assumption of non-articulating mesofemoral spines in *S. axelrodi*; (2) the assumption of damage to the mesotibial spines; (3) the assumption that mesofemoral spines are unique to *S. axelrodi*; and (4) the assumption that the presence of mesofemoral spines indicates a functional role in prey capture.

Hörnig, Haug & Haug (2013) list two morphological conditions of the mesothoracic appendages for known dictyopterans: (1) blattodeans bearing articulating short and blunt spines on the femora and uniformly arranged spines on the tibiae and (2) mantodeans lacking “prominent” femoral and tibial spination, noting extant species of *Chaeteessa* Burmeister, 1838 an exception as they bear articulating spines on the meso- and metathoracic legs, which is interpreted as the plesiomorphic state. The extinct mantis species *Cretomantis larvalis* Gratshev & Zherikhin, 1994 is another possible exception to condition 2; Grimaldi (2003) describes *C. larvalis* as bearing a mesofemur “with 2 ventral rows of spicules or minute spines.” Hörnig, Haug & Haug (2013) acknowledged that *C. larvalis* bears such mesofemoral spines, and subsequently determine that the species is an exception to condition 2 (i.e., it retains the plesiomorphic state). In a 2017 description of a new specimen of *S. axelrodi* MB.I.2068, Hörnig et al. state that the *C. larvalis* specimen described previously “appears to have born spines on the mesothoracic appendages, yet their exact nature is unclear,” adding that “in many instances spines appear to be broken off, preserving only the bases, with this hindering a clearer statement of the condition in this species.” They note that *C. larvalis* might possess mesothoracic leg spination morphology similar to *S. axelrodi* but ultimately, Hörnig, Haug & Haug (2017) do not conclusively determine which condition is present in *C. larvalis* due to its preservation (see Assumption 2 for continued discussion). Hörnig, Haug & Haug (2017) describe a third mesothoracic leg condition based on the spination observed on *S. axelrodi* MB.I.2068. The observed spines were described as “erect immovable prominent pointed,” and thus not as in condition 1 or 2.

## Results

### Assumption 1: mesofemoral spine non-articulation

**Figure 1:**
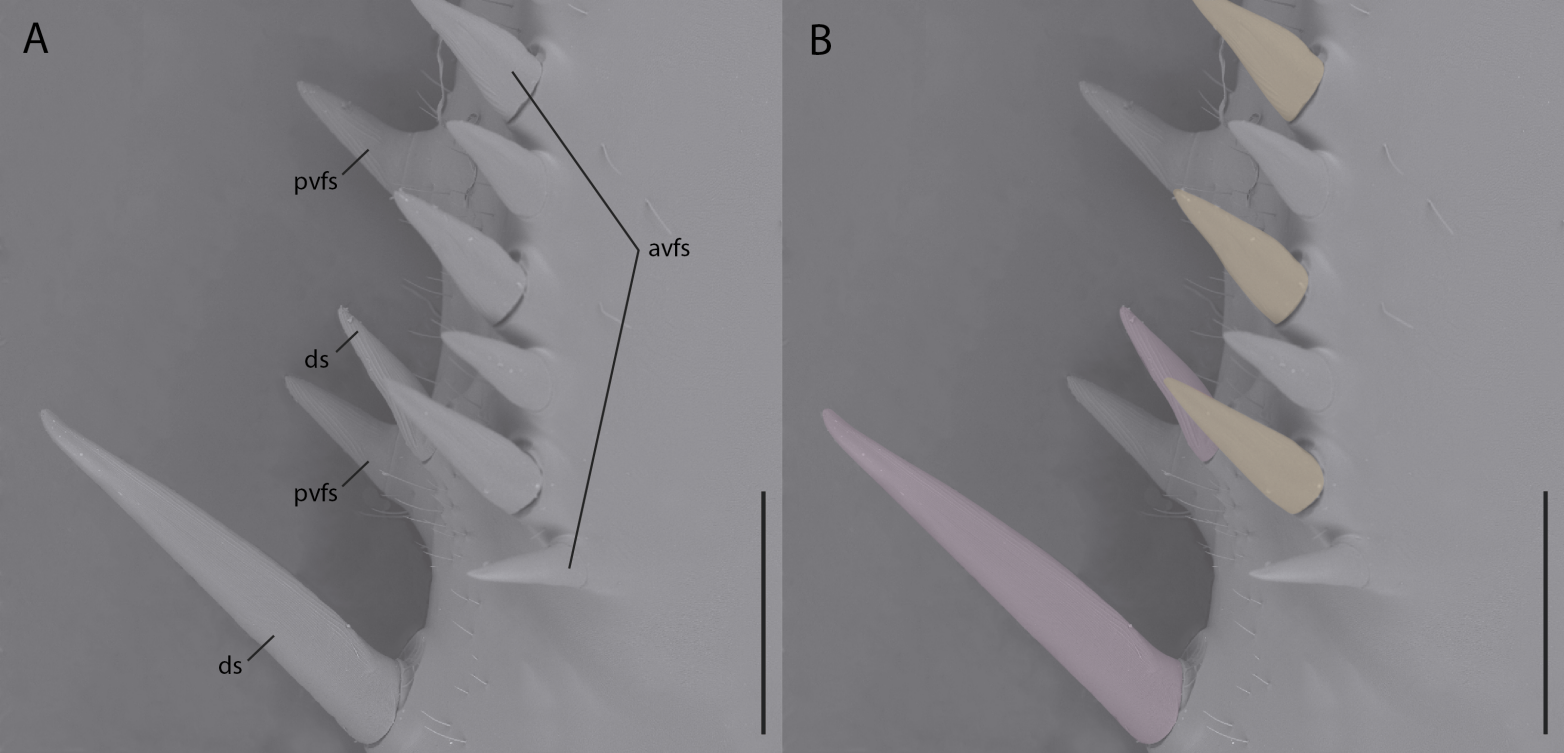
Environmental scanning electron micrograph (40×) of the prothoracic femur of *Tenodera sinensis* in anteroventral view. (A) Note the erectness, rigidity, and gradual proximal widening across all of the spines; (B) spines highlighted pink are articulating discoidal spines; spines highlighted orange are articulating anteroventral femoral spines; spines without highlighting do not articulate. Abbreviations: avfs, anteroventral femoral spines; ds, discoidal spines; pvfs, posteroventral femoral spines. Distracting debris was removed with the stamp and healing brush tools in Adobe Photoshop CC 2015. Scale bar = 1 mm.

While reconstructions of *Santanmantis axelrodi* MB.I.2068 clearly demonstrate the erectness, prominence, and pointedness of the spines, the articulating nature cannot be determined from the authors’ methodology. Based on the photographs of this specimen, as well as photographs and reconstructions of other *S. axelrodi* specimens (e.g., Grimaldi, 2003; Hörnig, Haug & Haug, 2013), there appears to be no obvious morphological features in the prothoracic spination or the mesothoracic spination to conclusively determine the articulatability of any of the observed spines. In modern mantises some prothoracic anteroventral and discoidal spines articulate (Wieland, 2013), which aid the tibial flexion reflex via proprioceptive feedback during prey capture (Copeland & Carlson, 1977; Prete, 1990); however, without the use of microscopes or high resolution macrophotography, the articulation point is difficult if not impossible to observe on preserved, non-fossilized specimens, let alone fossilized specimens that might be incompletely or poorly preserved. Hörnig, Haug & Haug (2017) provide no clear indication of the methodology they use to determine the articulatability of the observed spination, aside from writing that “there is no clear indication that these spines were jointed and movable; in contrast, they widen at the base, indicating their general rigidity.” As with the majority of mantodean spines, both articulating and non-articulating spines present on extant mantises widen at the base (e.g., *Tenodera* Burmeister, 1838, *Sphodromantis* Stål, 1871, *Stagmomantis* Saussure, 1869, *Hierodula* Burmeister, 1838, etc.) (Fig. 1) and so the given character state (e.g., spine widens at the base) does not conclusively determine the articulatability of such a spine and further, the absence of evidence of articulation does not indicate inarticulation.

### Assumption 2: mesotibial spine length and damage

Based on the photograph of the *Santanmantis axelrodi* MB.I.2068 fossil specimen presented in Hörnig, Haug & Haug (2017: fig. 1a), it is difficult to observe the proximal and medial mesothoracic tibial spines in the fossil specimen as they have depicted in the illustration (Hörnig, Haug & Haug, 2017: fig. 4), which features uniformly elongate, erect mesotibial spination. The authors state that the spines along the entire anteroventral edge (i.e., median edge *sensu* Hörnig, Haug & Haug, 2017) of the mesotibia have been “broken off close to the base” (Hörnig, Haug & Haug, 2017), and just as they themselves note, determining length, shape, and other morphological features of these spines cannot be estimated, rendering their illustration of the spines without evidentiary support (see Hörnig, Haug & Haug, 2017: fig. 4). Further, in this 2017 *S. axelrodi* reconstruction, the mesothoracic femoral spines do not appear to be represented as in the fossil specimen: in the reconstruction, all of the spines on the mesothoracic femur are uniform in length, however, based on the specimen presented in Hörnig, Haug & Haug (2017), the proximal mesothoracic femoral spines appear to be relatively longer than the distal spines. It is interesting to note that when Hörnig, Haug & Haug (2017) consider *Cretomantis larvalis* with mesothoracic spines that are apparently “broken off, preserving only the bases,” they err on the side of caution and do not conclusively determine which mesothoracic leg condition the specimen exhibits (i.e., the plesiomorphic condition or the third condition with erect, immobile spines). However, when the authors consider the *S. axelrodi* MB.I.2068 specimen, which features mesotibial spines “broken off close to the base,” they consider these spines, along with the spines observed on the mesofemora, to be “massive” and “prominent” and as representing the third condition, thus applying their analytical methodology inconsistently.

**Figure 2:**
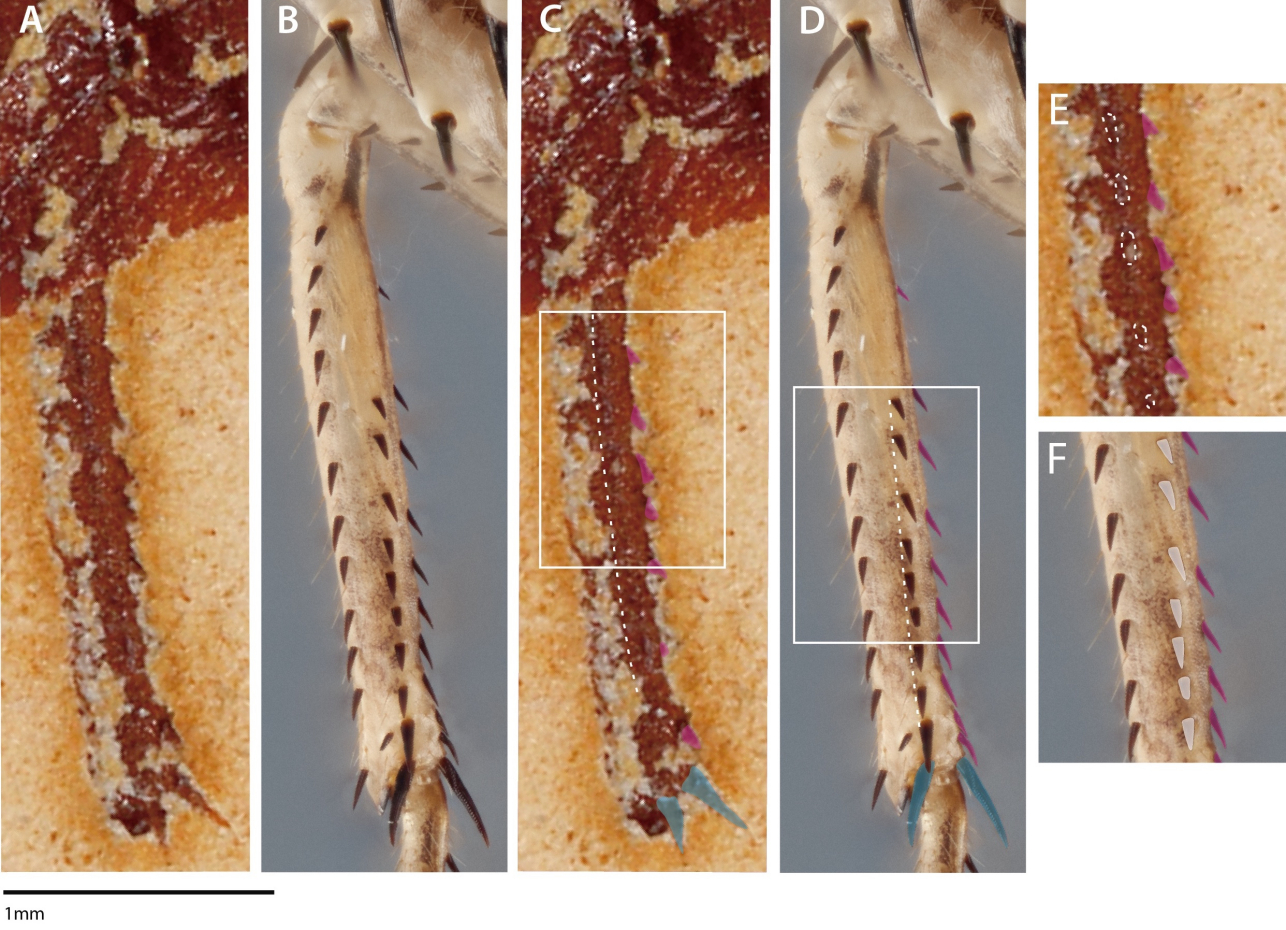
Mesothoracic tibiae of *Santanmantis axelrodi* MB.I.2068 (A, C, E) and *Chaeteessa* sp. (B, D, F) in ventral view. (A) *Santanmantis axelrodi* mesotibia; (B) *Chaeteessa* sp. mesotibia; (C) *S. axelrodi* mesotibial structures highlighted as in Hörnig, Haug & Haug (2017) with anteroventral spines in pink and apical tibial spurs in blue; white dashed line traversing the tibia demarcates apparent posteroventral structures; (D) *Chaeteessa* sp. mesotibial anteroventral spines highlighted pink and apical tibial spurs in blue; white dashed line traversing the tibia demarcates posteroventral structures topologically homologous to those observed in (C); (E) window showcases possible posteroventral spines observed in *S. axelrodi* outlined in white dashes; (F) window showcases posteroventral spines in *Chaeteessa* sp. highlighted in white. A, C, and E are reproduced and modified from Hörnig, Haug & Haug (2017). Photographs enhanced with the pen and rectangle tools in Adobe Illustrator CC 2015.

In both *S. axelrodi* specimens described by Hörnig, Haug & Haug (2013: AI 1736, 2017: MB.I.2068), one or two distal, elongated spines are clearly observable on the apex of the mesothoracic tibia. These apical mesothoracic tibial spurs (*n* = 2; tibial spur *sensu* Brannoch et al. (2017)) are always present on praying mantis taxa (Roy, 1999) and are not involved in extant mantodean prey capture. It is hard to ascertain why Hörnig, Haug & Haug (2017) assumed that the proximal and mesal mesotibial spines were broken when the apical mesotibal spurs and the mesofemoral spines are apparently well preserved; a more parsimonious explanation is to assume that the mesotibial spines are not broken. The specimen presented in Hörnig, Haug & Haug (2017) also appears to have a longitudinal, posteroventral mesotibial structure (e.g., a keel with spination) (Figs. 2A, 2C, 2E). This posteroventral mesotibial structure, when considered alongside the anteroventral spines, is strongly reminiscent of the cockroach-like spination present on *Chaeteessa* (Figs. 2B, 2D, 2F). This is in direct contradiction to the conclusions drawn by Hörnig, Haug & Haug (2017); they contend that the observed mesothoracic leg spination in *S. axelrodi* MB.I.2068 does not resemble in “structure and arrangement” the mesothoracic leg spination observed in *Chaeteessa*. Due to the inability to determine the articulatibility of the mesofemoral and -tibal spines based on the methodology presented in Hörnig, Haug & Haug (2017), condition 3 as erected by Hörnig, Haug & Haug (2017) may no longer be satisfied by *S. axelrodi* in that the characters are more like the plesiomorphic condition of *Chaeteessa*. We raise these concerns regarding the mesothoracic tibial spines as they present a potential problem in both the interpretation of mantodean character evolution and raptorial behavior presented in Hörnig, Haug & Haug (2017).

### Assumption 3: extant lineages without mesothoracic spines

**Figure 3:**
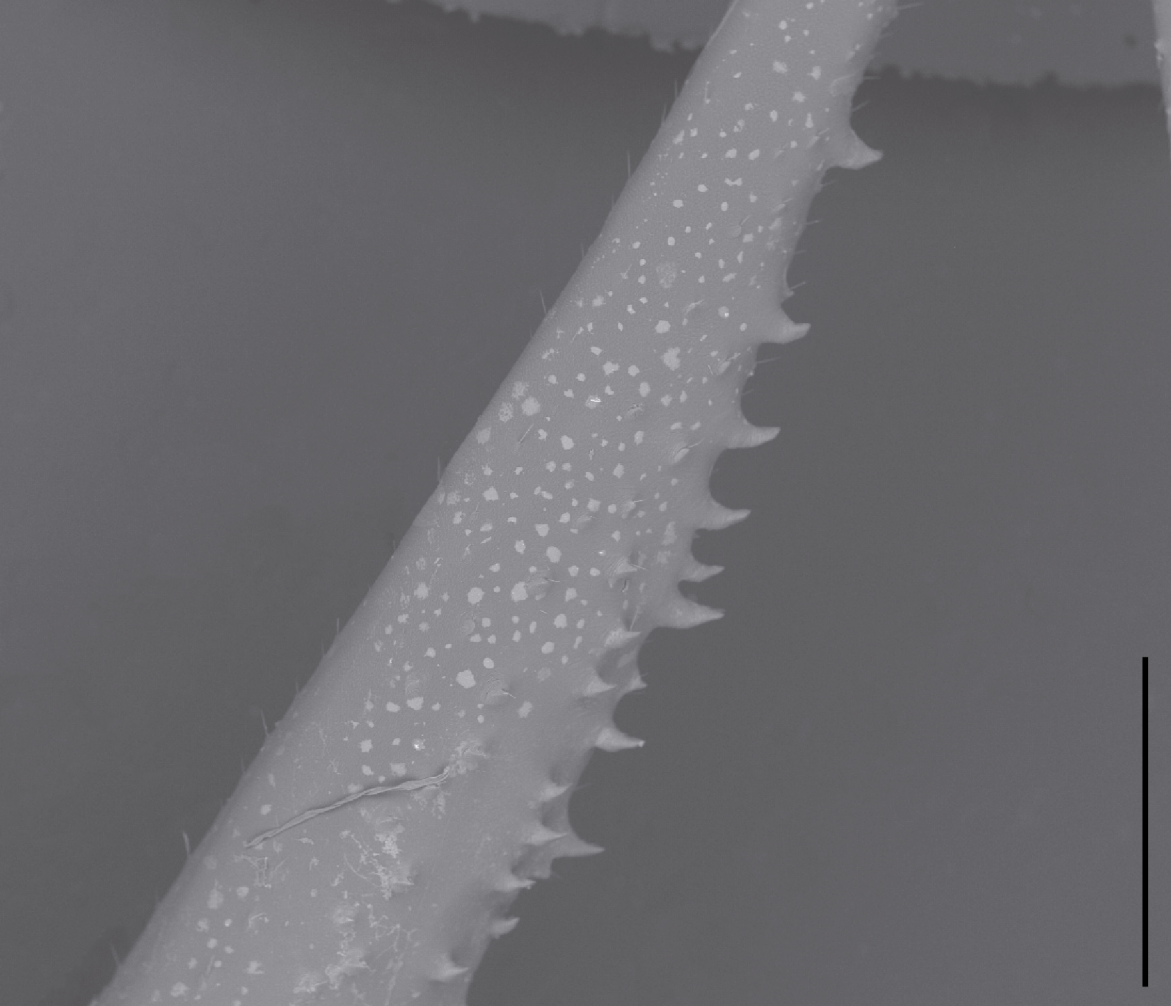
Environmental scanning electron micrograph (40×) of *Ciulfina* sp. mesothoracic femur. Note the immobile spines directed posteriorly along the anteroventral edge. Distracting debris was removed with the stamp and healing brush tools in Adobe Photoshop CC 2015. Scale bar = 1 mm.

Hörnig, Haug & Haug (2017) report there are no extant mantis species with non-articulating spines on the meso- and metathoracic legs, however the genera *Eremiaphila* Lefebvre, 1835, *Astape* Stål, 1877, *Metallyticus*, Westwood, 1835, and *Ciulfina* Giglio-Tos, 1915 possess such spines (Lieftinck, 1953; Ranade, Mukherjee & Ghate, 2004; Wieland, 2013). Some species of *Ciulfina* feature rows of immovable spines on their meso- and metathoracic legs, which are not used in hunting or immobilizing prey items (G Howell, pers. comm., 2017; S Brannoch, pers. obs., 2016) (Fig. 3). While in Wieland (2013), the aforementioned spines are not morphologically considered to be “true” spines in that they are cuticular outgrowths that do not feature a basal sulcus (see: Wieland (2013), figs 288–293), this does not necessarily mean that they are not “spines” in a functional sense (compare Figs. 1 and 3). A spine, as defined by Gordh & Headrick (2001) is “a stiff, sharp, pointed, tapered process on the surface of a plant or animal” or “a large seta provided with a calyx or cup by which it is articulated to the Cuticle...” Grimaldi (2003) defines spines as “socketed, sclerotized structures, slightly to considerably thicker than setae.” While there is a technical difference between a spine-like outgrowth (i.e., without a basal sulcus) and a true spine (i.e., with a basal sulcus), a spine in the general sense can functionally serve for defense, camouflage, predation, mechanoreception, etc. (French, 1984; Prete & Hamilton, 1999; Michaud & Grant, 2003; Wieland, 2013). This morphological distinction raises an issue: are the spines observed in *S. axelrodi* “true” or “functional” spines? Making such a determination with the presently available fossil material lands Hörnig et al. in a problematic situation similar to determining whether or not the fossilized spines of *S. axelrodi* could articulate. While these non-articulating, “functional” spines on the meso- and metathoracic appendages of *Eremiaphila*, *Astape*, *Metallyticus*, and *Ciulfina* do not necessarily diminish the “foreleg first” hypothesis (i.e., that forelegs specialized prior to reductions in spines on the meso- and metathoracic legs) detailed by Hörnig, Haug & Haug (2013) and Hörnig, Haug & Haug (2017), it is our view that by not considering the possession of spines on the mesothoracic legs of extant taxa, as well as committing methodological oversights in determining the mobility and precise morphology of the mesothoracic spines, their hypotheses on mantodean character evolution and predatory behavior are ultimately impacted.

### Assumption 4: spine presence indicative of prey capture function

The raptorial prothoracic legs of praying mantises are highly flexible, and can be raised in front of the body (Wieland, 2013), and, in the *S. axelrodi* reconstruction in question, the prothoracic legs appear to be treated as the cursorial legs in that they are stretched out to the side. Similar foreleg conditions have been observed to be present in other extant basal lineages, including *Chaeteessa*, *Mantoida*, and *Metallyticus*, and is likely a plesiomorphic condition (Wieland, 2013). Hörnig, Haug & Haug (2017) themselves note that early extant mantodeans rest with their prothoracic tarsi on substrate (for discussion see Hörnig, Haug & Haug, 2017: pg. 13), but when considered alongside the purported raptorial implications of possessing mesothoracic femoral and tibial spines, we contend that the authors treated the cursorial legs similar to the prothoracic legs in a *biomechanical* sense. Hörnig, Haug & Haug (2017) suggest that the mesothoracic spination “strongly indicates” that the mesothoracic appendages were involved in prey capture with members of *S. axelrodi* standing directly over prey, presumably using their mesofemoral and mesotibial spines to immobilize prey. It does not follow that the mere possession of spines on the mesothoracic appendages is indicative of a predation strategy incorporating such spines; it is merely indicative of the possession of such spines.

The spines present on the mesothoracic legs is a red herring, distracting from the biomechanical aspect of prey handling. The true issue lies in the authors’ implicit assumption about the level of mobility achievable by the mesothoracic legs if they are used for predation. This assumption is not corroborated by any evidence present in the fossil specimens or in early modern mantises, which do not possess such mobility. Given the morphology of the mesothoracic legs observed in *S. axelrodi* specimens, we do not see any evidence for increased mobility in comparison to early extant species. As Hörnig, Haug & Haug (2017) state that early dictyopteran lineages resemble modern cockroaches and further, that early mantodeans are presumed to be active hunters similar to *Metallyticus*, there is no reason to consider the meso- and metathoracic legs of *S. axelrodi* to have an alternate function. Contrary to Hörnig, Haug & Haug (2017) and Lee (2014) described specimens of *S. axelrodi* as featuring long mid- and hindlegs “with retained cursorial function.” Imagine a living *S. axelrodi* specimen: if it were to capture and immobilize a passing insect in the way that Hörnig, Haug & Haug (2017) are suggesting, either (1) the mesothoracic legs would have to rotate anteriorly, straighten, and compress against each other, thereby entrapping prey, or (2) the mesothoracic legs would have to “hug” a prey item against the body with the dextral and sinistral mesotibiae positioned underneath. In both cases, the specimen’s posture and balance would be greatly impaired as they would no longer be relying on both the meso- and metathoracic legs for maintaining contact with the ground. Further, while it is known that praying mantises can strike at prey anterior to, near, and directly below their head capsules (Prete & Hamilton, 1999), the method posited by Hörnig, Haug & Haug (2017) would require *S. axelrodi* to rely on a biomechanically less effective set of legs to immobilize prey, while still necessitating the use of the raptorial forelegs for prey consumption.

## Conclusion

We conclude that the spines present on fossil *Santanmantis axelrodi* specimens were most likely not used in prey capture or prey immobilization, contrary to the conclusions of Hörnig, Haug & Haug (2017). While the possibility of mesothoracic prey capture is at best speculative, it cannot be excluded, which is an unavoidable deficiency in paleobiology. With such mesofemoral morphology and spination present in outgroup taxa and interpreted as plesiomorphic by Grimaldi (2003), it is ill advised to attribute a novel hunting strategy to a fossil taxon that does not diverge significantly from known morphologies in both extinct and extant lineages. Hörnig, Haug & Haug (2017) use an antiquated system of evolutionary systematics, in which they interpret the phylogenetic position of taxa and the evolution of character transitions based on the actual characters being investigated exclusive of a formal analysis. Problems of circularity inherent to this methodology are compounded by the exclusion of relevant fossil taxa that retain the characters that Hörnig, Haug & Haug (2017) used to interpret their cladogram (e.g., *Cretomantis* Gratshev & Zherikhin, 1994). Hörnig, Haug & Haug (2017) also appear to pick and choose mantodean and insect correlates that positively apply to their interpretations of *S. axelrodi* biology and behavior. Ultimately they determine that there are no comparable insect correlates that exhibit similar morphology to *S. axelrodi* and hunt with the mesothoracic legs. However, investigating Mantodea in a broader context reveals exceptions to their conclusions based on these correlates. Their attribution of a novel hunting strategy in *S. axelrodi* is based on the absence of observation rather than the presence of corresponding morphological and behavioral characters within the insect correlates that they consider. Therefore, we contend that the methodological limitations, inadequate taxonomic comparison, and poorly justified assumptions preclude the evolutionary interpretations made by Hörnig, Haug & Haug (2017). *Santanmantis axelrodi* was an early praying mantis species that most likely employed the “normal” set of praying mantis behaviors, not a species with a novel hunting strategy relative to other Mantodea.

## Additional Information and Declarations

### Data Availability

The following information was supplied regarding data availability: The raw data is included in the Figures in the manuscript.

## References

[ref-1] Brannoch SK, Wieland F, Rivera J, Klass K-D, Béthoux O, Svenson GJ (2017). Manual of praying mantis morphology, nomenclature, and practices (Insecta, Mantodea). ZooKeys.

[ref-2] Copeland J, Carlson AD (1977). Prey capture in mantids: prothoracic tibial flexion reflex. Journal of Insect Physiology.

[ref-3] French AS (1984). Action potential adaptation in the femoral tactile spine of the cockroach, *Periplaneta americana*. Journal of Comparative Physiology A: Neuroethology, Sensory, Neural, and Behavioral Physiology.

[ref-4] Gordh G, Headrick DH (2001). A dictionary of entomology.

[ref-5] Grimaldi D (2003). A revision of Cretaceous mantises and their relationships, including new taxa (Insecta: Dictyoptera: Mantodea). American Museum Novitates.

[ref-6] Hörnig MK, Haug JT, Haug C (2013). New details of *Santanmantis axelrodi* and the evolution of the mantodean morphotype. Palaeodiversity.

[ref-7] Hörnig MK, Haug JT, Haug C (2017). An exceptionally preserved 110 million years old praying mantis provides new insights into the predatory behaviour of early mantodeans. PeerJ.

[ref-8] Klass K-D, Ehrmann R, Kaestner A (2003). Lehrbuch der speziellen Zoologie. Wirbellose Tiere. Spezieller Teil: Insecta - Mantodea (Hrsg. H H Dathe), 2. Aufl., 1(5).

[ref-9] Lee SW (2014). New lower cretaceous basal mantodean (Insecta) from the Crato Formation (NE Brazil). Geologica Carpathica.

[ref-10] Lieftinck MA (1953). Biological and ecological observations on a bark hunting mantid in Java (Orthopt., Mantoidea). Transactions of the Ninth International Congress of Entomology.

[ref-11] Michaud JP, Grant AK (2003). Intraguild predation among ladybeetles and a green lacewing: do the larval spines of *Curinus coeruleus* (Coleoptera: Coccinellidae) serve a defensive function?. Bulletin of Entomological Research.

[ref-12] Prete FR (1990). Prey capture in mantids: the role of the prothoracic tibial flexion reflex. Journal of Insect Physiology.

[ref-13] Prete FR, Hamilton, Prete FR, Wells H, Wells PH, Hurd LE (1999). The praying mantids. Prey capture.

[ref-14] Ranade SP, Mukherjee S, Ghate HV (2004). A note on desert mantis *Eremiaphila rotundipennis* Kirby (Insecta: Mantodea: Eremiaphilidae) from Rajasthan, India. Zoos’print Journal.

[ref-15] Roy R, Prete FR, Wells H, Wells PH, Hurd LE (1999). The praying mantids. Morphology and taxonomy.

[ref-16] Wieland F (2013). The phylogenetic system of Mantodea (Insecta: Dictyoptera).

